# An Automatic Calibration Method for Kappa Angle Based on a Binocular Gaze Constraint

**DOI:** 10.3390/s23083929

**Published:** 2023-04-12

**Authors:** Jiahui Liu, Jiannan Chi, Hang Sun

**Affiliations:** 1 School of Automation and Electrical Engineering, University of Science and Technology Beijing, Beijing 100083, China; 2 Beijing Engineering Research Center of Industrial Spectrum Imaging, University of Science and Technology Beijing, Beijing 100083, China; 3 Shunde Innovation School, University of Science and Technology Beijing, Foshan 528399, China

**Keywords:** kappa angle, user calibration, binocular constraint, gaze estimation, eye-tracking interaction

## Abstract

Kappa-angle calibration shows its importance in gaze tracking due to the special structure of the eyeball. In a 3D gaze-tracking system, after the optical axis of the eyeball is reconstructed, the kappa angle is needed to convert the optical axis of the eyeball to the real gaze direction. At present, most of the kappa-angle-calibration methods use explicit user calibration. Before eye-gaze tracking, the user needs to look at some pre-defined calibration points on the screen, thereby providing some corresponding optical and visual axes of the eyeball with which to calculate the kappa angle. Especially when multi-point user calibration is required, the calibration process is relatively complicated. In this paper, a method that can automatically calibrate the kappa angle during screen browsing is proposed. Based on the 3D corneal centers and optical axes of both eyes, the optimal objective function of the kappa angle is established according to the coplanar constraint of the visual axes of the left and right eyes, and the differential evolution algorithm is used to iterate through kappa angles according to the theoretical angular constraint of the kappa angle. The experiments show that the proposed method can make the gaze accuracy reach 1.3${}^{{}^{\circ}}$ in the horizontal plane and 1.34${}^{{}^{\circ}}$ in the vertical plane, both of which are within the acceptable margins of gaze-estimation error. The demonstration of explicit kappa-angle calibration is of great significance to the realization of the instant use of gaze-tracking systems.

## 1. Introduction

Gaze tracking aims to determine the user’s current gaze direction or gaze point from facial or eye features, and realize automatic interaction and attention analysis by analyzing the user’s eye movement information. At present, gaze tracking has been applied to an eye-control mouse [1], a virtual reality helmet [2], disease screening and diagnosis [3], and attention analysis [4], which relates to human–computer interaction, virtual reality, wise medicine, etc. It has a broad application prospect.

The core of gaze tracking is gaze estimation. According to the eyeball structure, the optical axis (OA) of the eyeball passes through the eyeball center, the corneal center, the iris center and the pupil center, and is perpendicular to the iris or pupil plane. The visual axis (VA) of the eyeball passes through the fovea and the corneal center. There is a fixed deflection angle between the OA and the VA called the kappa angle [5]. Three-dimensional gaze estimation refers to the estimation of the VA of the eyeball. However, the VA cannot be determined only by the corneal center due to the invisibility of the fovea. The general method is to reconstruct the OA of the eyeball first, and then use the kappa angle to generate the VA from the OA. The kappa angle has individual differences, and it is not universal practice to set it with a fixed value. Therefore, the calibration of kappa angle is especially significant, which will directly affect the gaze accuracy.

The existing kappa-angle-calibration methods are mainly explicit, requiring the user to look at some pre-defined calibration points on the screen before eye-gaze tracking. During this explicit calibration process, the user’s eye-feature parameters are extracted from the collected facial images, and the OA and the VA of the eyeball when the user looks at each calibration point can be calculated to represent the kappa angle. The kappa angle has two possible representations. One is represented by the transformation matrix between the OA and the VA. The researchers calibrated the transformation matrix using multiple groups of OA directions and their corresponding VA directions [6,7]. This method usually uses multiple calibration points to ensure the robustness of the calibrated transformation matrix, but multi-point user calibration determines the complexity of the calibration process. To simplify the calibration, some researchers put forward the method of representing the kappa angle using angles. In the eyeball coordinate system, both the OA direction and the VA direction can be represented by a trigonometric function of a horizontal angle and a vertical angle. The horizontal and vertical angles of VA direction can be regarded as the superposition of the horizontal and vertical angles of OA direction and the kappa angle. Thus, the horizontal and vertical components of kappa angle can be calculated using a single calibration point [8,9,10,11,12,13]. In addition, a few researchers used one or two angles to represent the transformation angle between the OAs of the eyeball at two different positions, thereby calculating the VA direction of the eyeball at one position using the VA direction at another position and the transformation angle, since the positional relationship between the OA and the VA at different positions remains unchanged [14,15,16]. Xiong et al. [17] used the horizontal and vertical rotation angles from the OA to the VA in a camera coordinate system to represent the transformation matrices for the horizontal and vertical directions. The product of these two matrices was then used to represent the kappa angle. Wan et al. [18] calculated the horizontal- and vertical-rotation matrices between the OA and the VA, and determined the horizontal and vertical components of the kappa angle by averaging the horizontal and vertical rotation angles obtained by looking at each calibration point. However, the explicit calibration method increases the complexity of the system’s use, which is contrary to the "instant use" pursuit of natural human–computer interaction. Although the number of calibration points can be reduced to one, multi-point calibration seems to be more robust than single-point calibration.

To improve the actualities, Nagamatsu et al. [19] approximated the midpoint of the intersections of the OAs of both eyes and the screen as the point of regard (POR), so they estimated the horizontal angle of the kappa angle without requiring user calibration. However, the vertical component is not considered, and the difference in kappa angle between left and right eyes is ignored. Wang and Ji [20] used four staring constraints to formulate an implicit kappa-angle calibration as a constrained unsupervised regression problem and solved it through an iterative hard EM algorithm. This method is friendly to users, but the distance between the eyeball center and the corneal center is set as a constant when calculating the eyeball’s center, which will lead to different deviations in the 2D mapping model for different users, resulting in an equal offset between the 2D POR and the 3D POR.

In this paper, an automatic calibration method of kappa angle based on the binocular gaze constraint is proposed. An optimal objective function of the kappa angle is established by using the coplanar constraint of the VAs of the left and right eyes. Additionally, the differential evolution algorithm is used to iterate toward the optimal solution of the kappa angle within its theoretical range.

The main contributions of this paper are:

(1) The automatic calibration of the kappa angle is realized by using the binocular gaze constraint that the VAs of the left and right eyes should be coplanar. It avoids the explicit calibration of the kappa angle while both individual differences and the differences between left and right eyes are considered.
(2) The differential evolution algorithm is used to solve the established optimal objective function of the kappa angle within its theoretical range, which makes the kappa-angle calibration accurate. It avoids the setting of the initial value in the conventional solution method, as the kappa-angle calibration is susceptible to the initial value.

The rest of this paper is organized as follows. Section 2 discusses the existing calibration methods for the kappa angle. Section 3 describes the proposed automatic calibration method for the kappa angle in detail. The experiments involving computer simulations and practical experiments are analyzed in Section 4. Section 5 summarizes and concludes the work.

## 2. Representation of Kappa-Angle Calibration

The kappa angle is the angle between the OA and the VA of the eyeball. Since the VA is the real gaze, the kappa angle is an essential parameter for estimating the 3D gaze. It has two representations: a matrix representation and an angular representation. They are discussed separately below.

### 2.1. Matrix Representation

**Figure 1:**
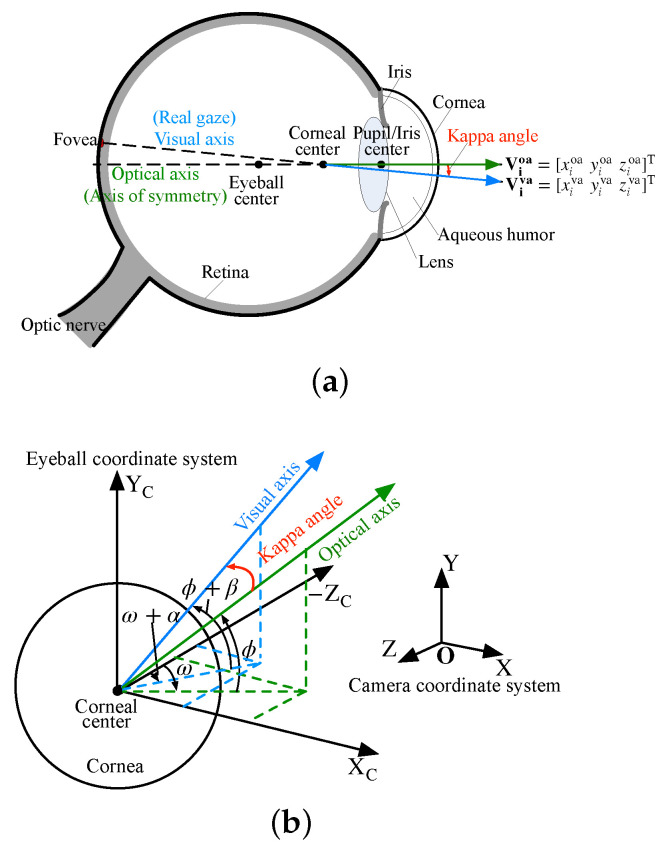
Kappa-angle representations. (**a**) Using a transformation matrix. (**b**) Using a horizontal angle and a vertical angle.

As Figure 1a shows, when the user looks at the *i*th calibration point, the unit-direction vector of the OA is expressed as $V_{i}^{\mathrm{oa}}={\left[\begin{array}{c} x_{i}^{oa}\;y_{i}^{oa}\;z_{i}^{oa} \end{array}\right]}^{T}$, and the unit-direction vector of the VA is expressed as $V_{i}^{\mathrm{va}}={\left[\begin{array}{c} x_{i}^{va}\;y_{i}^{va}\;z_{i}^{va} \end{array}\right]}^{T}$. They can be obtained via a multi-point user calibration process. Assume that the number of calibration points is *N*, and the size of both the OA matrix $V^{\mathrm{oa}}$ and the VA matrix $V^{\mathrm{va}}$ is 3×N. Thus, the relationship between the OA and the VA can be expressed by a transformation matrix M, whose size is 3×3. That is,

(1) $$V^{\mathrm{va}}=MV^{\mathrm{oa}}.$$

where $M=\left[\begin{array}{c} a_{1}\;a_{2}\;a_{3}\\ a_{4}\;a_{5}\;a_{6}\\ a_{7}\;a_{8}\;a_{9} \end{array}\right]$, $V^{\mathrm{oa}}=\left[\begin{array}{c} x_{1}^{oa}\;x_{2}^{oa}\;\ldots \;x_{N}^{oa}\\ y_{1}^{oa}\;y_{2}^{oa}\;\ldots \;y_{N}^{oa}\\ z_{1}^{oa}\;z_{2}^{oa}\;\ldots \;z_{N}^{oa} \end{array}\right]$, and $V^{\mathrm{va}}=\left[\begin{array}{c} x_{1}^{va}\;x_{2}^{va}\;\ldots \;x_{N}^{va}\\ y_{1}^{va}\;y_{2}^{va}\;\ldots \;y_{N}^{va}\\ z_{1}^{va}\;z_{2}^{va}\;\ldots \;z_{N}^{va} \end{array}\right]$. The coefficients $a_{1}∼a_{9}$ in M can be calculated using at least three calibration points. When more calibration points are used, the robustness of the transformation matrix M is stronger.

### 2.2. Angular Representation

As Figure 1b shows, an eyeball coordinate system is defined as follows: (1) the corneal center is the origin; (2) the *x*- and *y*-axes are the same as the horizontal and vertical directions of the screen; (3) the *z*-axis is perpendicular to the screen and points to the eyeball. In this coordinate system, the unit-direction vector of the OA can be expressed by a horizontal angle, ω, and a vertical angle, ϕ:

(2) $${\tilde{V}}^{\mathrm{oa}}=\left[\begin{array}{c} \cos\phi \sin\omega \\ \sin\phi \\ -\cos\phi \cos\omega \end{array}\right].$$

The kappa angle is decomposed into a horizontal angle, α, and a vertical angle, β. The average value of α is about 5${}^{{}^{\circ}}$, and the average value of β is about 1.5${}^{{}^{\circ}}$ [8]. With the OA direction and the kappa angle, the unit-direction vector of the VA is:

(3) $${\tilde{V}}^{\mathrm{va}}=\left[\begin{array}{c} \cos(\phi +\beta )\sin(\omega +\alpha )\\ \sin(\phi +\beta )\\ -\cos(\phi +\beta )\cos(\omega +\alpha ) \end{array}\right].$$

As the gaze point is known by explicit user calibration, the unit-direction vector of the VA in the camera coordinate system, represented by $V^{\mathrm{va}}$, is known. Thus, ${\tilde{V}}^{\mathrm{va}}$ can be obtained via the transformation between the camera coordinate system and the eyeball coordinate system. When ${\tilde{V}}^{\mathrm{va}}$ is substituted into Equation (3), α and β can be calculated. The kappa angle can be calibrated by using a single calibration point.

## 3. Proposed Method

**Figure 2:**
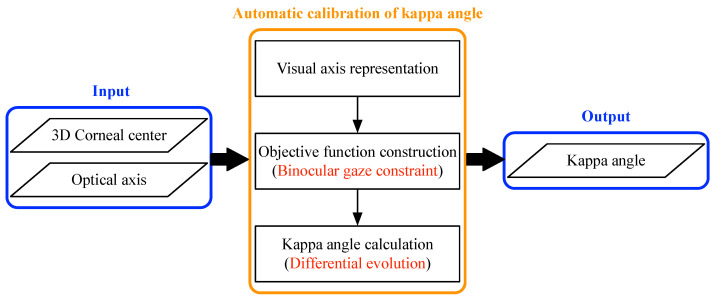
Procedure of the proposed method.

When calibrating the kappa angle, most existing methods require the user to look at some pre-defined calibration points. This explicit calibration is time-consuming and unfriendly. Although a few implicit calibration or calibration-free methods have been proposed [19,20], these methods do not fully consider the individual differences and the differences between the left and right eyes. Therefore, this paper proposes an automatic calibration method for the kappa angle. The basic procedure is shown in Figure 2. In the gaze-tracking system, based on the calculation of multiple groups of eyeball parameters that look at different positions, including the 3D corneal center and OA direction, the VA direction is represented by the kappa angle to be solved. An optimal objective function of the kappa angle is then constructed by using the binocular gaze constraint. Finally, the kappa angle is optimized and iterated using the differential evolution algorithm. The calibrated kappa angle can be used for subsequent real-time 3D gaze estimation.

### 3.1. Visual-Axis Representation

**Figure 3:**
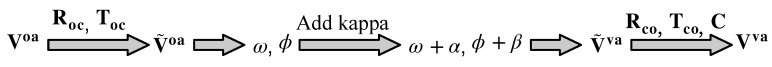
Visual-axis representation using kappa angle.

As mentioned in Section 2.2, angular representation is used to calibrate the kappa angle in this paper. Therefore, the representation of the VA mainly uses the transformation between the camera coordinate system and the eyeball coordinate system, as shown in Figure 3. The 3D corneal center (C) and the OA direction ($V^{\mathrm{oa}}$) in the camera coordinate system can be calculated according to the geometric imaging model of the eyeball. Using the coordinates of the screen points and the 3D corneal center, the transformation matrices ($R_{\mathrm{oc}}$, $T_{\mathrm{oc}}$) from the camera coordinate system to the eyeball coordinate system can be obtained, and the transformation matrices ($R_{\mathrm{co}}$, $T_{\mathrm{co}}$) from the eyeball coordinate system to the camera coordinate system can also be obtained. Thus, the OA direction (${\tilde{V}}^{\mathrm{oa}}$) in the eyeball coordinate system can be converted from $V^{\mathrm{oa}}$, and thereby ω and ϕ are calculated by substituting ${\tilde{V}}^{\mathrm{oa}}$ into Equation (2). The horizontal and vertical components of kappa angle are α and β, so the horizontal and vertical components of VA direction are (ω+α) and (ϕ+β). The representation of VA direction in the eyeball coordinate system is shown in Equation (3). The VA direction ($V^{\mathrm{va}}$) in the camera coordinate system can be represented using $R_{\mathrm{co}}$, $T_{\mathrm{co}}$, and C. The VA of the eyeball is related to the OA direction, the 3D corneal center, and the kappa angle—that is:

(4) $$V^{\mathrm{va}}=h\left(V^{\mathrm{oa}},C;\alpha ,\beta \right).$$

### 3.2. Objective Function Construction Using the Binocular Gaze Constraint

**Figure 4:**
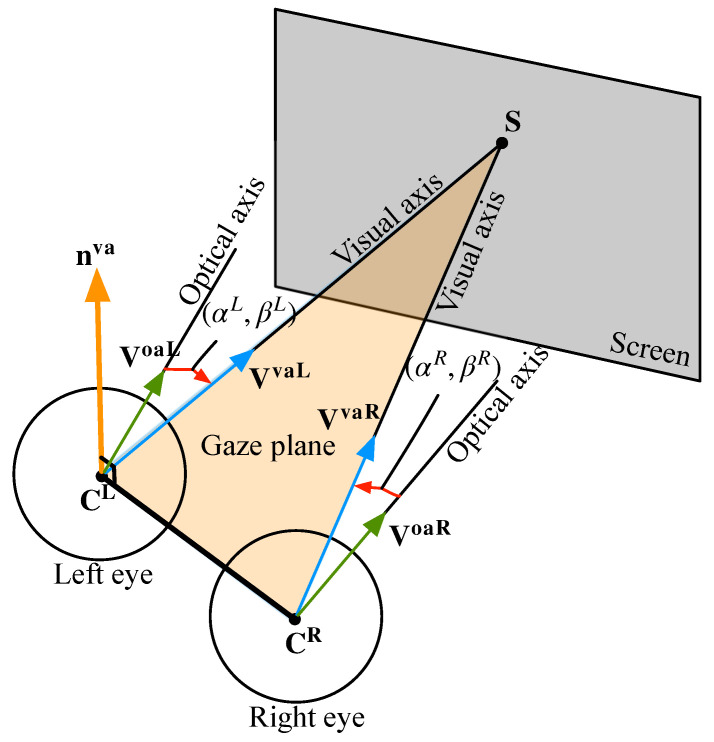
Binocular gaze constraint.

When a user gazes at the screen, ideally, the VAs of both eyes would intersect at a same point on the screen plane [21]. However, due to the presence of dominant and auxiliary eyes, the case that two VAs intersect at one point is not absolute. Therefore, a binocular gaze constraint is proposed in this paper, as shown in Figure 4. It does not require the explicit calibration points.

The horizontal and vertical components of the kappa angle of the left eye are expressed by $\alpha^{L}$ and $\beta^{L}$, and those of the right eye are expressed by $\alpha^{R}$ and $\beta^{R}$, so the VA directions of the left and right eyes are $V^{\mathrm{vaL}}=h\left(V^{\mathrm{oaL}},C^{L};\alpha^{L},\beta^{L}\right)$ and $V^{\mathrm{vaR}}=h\left(V^{\mathrm{oaR}},C^{R};\alpha^{R},\beta^{R}\right)$. Compared to the constraint that the VAs of the left and right eyes intersect at a point, the coplanarity of the VAs of the left and right eyes is used. We defined a gaze plane formed by the VAs of the left and right eyes, and the normal vector of the plane can be expressed by the VA directions of the left and right eyes. That is:

(5) $$n^{\mathrm{va}}=\frac{V^{\mathrm{vaL}}\times V^{\mathrm{vaR}}}{∥V^{\mathrm{vaL}}\times V^{\mathrm{vaR}}∥}.$$

The structure of the eyeball shows that the corneal center is on the VA of the eyeball, so the 3D corneal centers of the left and right eyes are both in the gaze plane, so the gaze plane can be determined uniquely. It satisfies:

(6) $$n^{\mathrm{va}}•\left(C^{L}-C^{R}\right)=0.$$

Therefore, based on the invariance of the kappa angle and the coplanar constraint of the VAs of the left and right eyes, an objective function is constructed. That is,

(7) $$\left[\alpha^{L*},\beta^{L*},\alpha^{R*},\beta^{R*}\right]=\arg\min\sum_{i=1}^{N}{\left(n_{i}^{\mathrm{va}}•\left(C_{i}^{L}-C_{i}^{R}\right)\right)}^{2}.$$

*N* is the amount of data for a user. The normal vector of the gaze plane of the *i*th group is $n_{i}^{\mathrm{va}}=\frac{V_{i}^{\mathrm{vaL}}\times V_{i}^{\mathrm{vaR}}}{∥V_{i}^{\mathrm{vaL}}\times V_{i}^{\mathrm{vaR}}∥}$, in which the VA direction of the left eye is $V_{i}^{\mathrm{vaL}}=h\left(V_{i}^{\mathrm{oaL}},C_{i}^{L};\alpha^{L},\beta^{L}\right)$, and that of the right eye is $V_{i}^{\mathrm{vaR}}=h\left(V_{i}^{\mathrm{oaR}},C_{i}^{R};\alpha^{R},\beta^{R}\right)$. To solve four unknowns ($\alpha^{L}$, $\beta^{L}$, $\alpha^{R}$, $\beta^{R}$), at least four groups of data are required to satisfy the coplanarity of the VAs of the left and right eyes; otherwise, Equation (7) is underdetermined, so *N* needs to be no less than 4. When more samples are used, the robustness of kappa-angle calibration will be stronger.

### 3.3. Kappa-Angle Calculation Using Differential Evolution Algorithm

The kappa angle can be accurately solved by the above objective function in ideal conditions. However, in the real scene, some errors in system or eyeball parameters may cause a large deviation between the optimal solution and the ground truth, or the solution is closely related to the initially set value. Therefore, the differential evolution algorithm is used in this paper to solve the kappa angle.

First, multiple groups of initial solution vectors are randomly generated in the form of $x=[\alpha^{L},\beta^{L},\alpha^{R},\beta^{R}]$ within the theoretical range of kappa angles, and the element of each solution vector satisfies:

(8) $$x_{k,j}=x_{k,j}^{E}+\left(x_{k,j}^{F}-x_{k,j}^{E}\right)*rand\left(\right).$$

where k=1,2,⋯,W, and j=1,2,3,4. *W* is the total number of solution vectors. $x_{k,j}$ refers to $\alpha^{L},\beta^{L},\alpha^{R},\beta^{R}$ in the *k*th solution vector, and their upper and lower limits are expressed by $x_{k,j}^{F}$ and $x_{k,j}^{E}$·rand() represents a random number generated between [0, 1].

For the above solution vectors, we first calculate the gaze-constraint error as shown in Equation (9). A solution vector that minimizes the gaze-constraint error is taken as the initial optimal kappa angle, and its gaze-constraint error is taken as the initial error for subsequent comparison.

(9) $$f\left(x\right)=\sum_{i=1}^{N}{\left(\frac{h\left(V_{i}^{\mathrm{oaL}},C_{i}^{L};x\left[0\right],x\left[1\right]\right)\times h\left(V_{i}^{\mathrm{oaR}},C_{i}^{R};x\left[2\right],x\left[3\right]\right)}{∥h\left(V_{i}^{\mathrm{oaL}},C_{i}^{L};x\left[0\right],x\left[1\right]\right)\times h\left(V_{i}^{\mathrm{oaR}},C_{i}^{R};x\left[2\right],x\left[3\right]\right)∥}•\left(C_{i}^{L}-C_{i}^{R}\right)\right)}^{2}.$$

Three different solution vectors from the above solution vectors are then used to generate the mutated solution vector. The element of the mutated solution vector satisfies:

(10) $$v_{k,j}=x_{r_{1},j}+K*\left(x_{r_{2},j}-x_{r_{3},j}\right).$$

where $1\leq r_{1},r_{2},r_{3}\leq W$, and $k\neq r_{1}\neq r_{2}\neq r_{3}$. *K* is the mutation operator. If $v_{k,j}$, calculated by Equation (10), exceeds the upper and lower limits we set, $v_{k,j}$ takes the boundary value.

Using the initial solution vector $x_{k}$ and the mutated solution vector $v_{k}$, the crossover operation is performed to generate a new solution vector $u_{k}$, as Equation (11) shows. Whether $u_{k}$ is $x_{k}$ or $v_{k}$ depends on the comparison between a crossover operator *c* and a random number.

(11) $$\begin{array}{c} u_{k}=\left\{\begin{array}{c} v_{k},\;rand\left(\right)\leq c\\ x_{k},\;rand\left(\right)>c \end{array}\right.. \end{array}$$

The gaze-constraint errors for $x_{k}$ and $u_{k}$ are then, respectively, calculated using Equation (9). The final solution vector $X_{k}$ for the comparison is represented by the one that makes the gaze-constraint error smaller. That is:

(12) $$\begin{array}{c} X_{k}=\left\{\begin{array}{c} u_{k},\;f\left(u_{k}\right)<f\left(x_{k}\right)\\ x_{k},\;f\left(u_{k}\right)\geq f\left(x_{k}\right) \end{array}\right.. \end{array}$$

After obtaining $X_{k}$, the gaze-constraint errors corresponding to $X_{1}$, $X_{2}$, ..., $X_{W}$ are calculated. The solution vector that minimizes the gaze-constraint error is taken as the optimal solution of this iteration, as shown in Equation (13).

(13) $$X^{*}=\arg\min f\left(X_{k}\right),\;k=1,2,\ldots ,W.$$

The iteration stops when the gaze-constraint error is less than the iteration termination error, and the solution vector at this time is used as the calibration result of the kappa angle. The specific calculation process is shown in Algorithm 1.

**Algorithm 1:** Process of kappa-angle calculation.
**Input:** 
3D corneal centers of left and right eyes ($C^{L}$ and $C^{R}$), OA directions of left and right eyes ($V^{\mathrm{oaL}}$ and $V^{\mathrm{oaR}}$), number of solution vectors *W*, upper and lower limits of kappa angle ($x_{k,j}^{F}$ and $x_{k,j}^{E}$), upper limit of iterations *T*, mutation operator *K*, crossover operator *c*, iteration termination error δ;**Output:** 
Optimal kappa angle $\left[\alpha^{L*},\beta^{L*},\alpha^{R*},\beta^{R*}\right]$;1:**for** k=1 to *W* **do**2:    $x_{k,j}=x_{k,j}^{E}+\left(x_{k,j}^{F}-x_{k,j}^{E}\right)*rand\left(\right),\;x_{k,1},x_{k,2},x_{k,3},x_{k,4}=\alpha^{L},\beta^{L},\alpha^{R},\beta^{R}$;3:
**end for**
4:$x^{\mathrm{op}}=\arg\min f\left(x_{k}\right),\;k=1,2,...,W$;5:$\epsilon^{op}=f\left(x_{\mathrm{op}}\right)$;6:**for** t=1 to *T* **do**7:    **for** k=1 to *W* **do**8:        $v_{k,j}=x_{r_{1},j}+K*\left(x_{r_{2},j}-x_{r_{3},j}\right),\;1\leq r_{1},r_{2},r_{3}\leq W,\;k\neq r_{1}\neq r_{2}\neq r_{3}$;9:        **if** $v_{k,j}<x_{k,j}^{E}$ **then**10:           $v_{k,j}=x_{k,j}^{E}$;11:        **else if** $v_{k,j}>x_{k,j}^{F}$ **then**12:           $v_{k,j}=x_{k,j}^{F}$;13:        **end if**14:        **if** rand()≤c **then**15:           $u_{k}=v_{k}$;16:        **else**17:           $u_{k}=x_{k}$;18:        **end if**19:        **if** $f\left(u_{k}\right)<f\left(x_{k}\right)$ **then**20:           $X_{k}=u_{k}$;21:        **else**22:           $X_{k}=x_{k}$;23:        **end if**24:        **if** $f\left(X_{k}\right)<\epsilon^{op}$ **then**25:           $\epsilon^{op}=f\left(X_{k}\right)$;26:           $x_{t}^{\mathrm{op}}=X_{k}$;27:        **end if**28:    **end for**29:    **if** $f(x_{t}^{\mathrm{op}})<\delta$ **then**30:        $\left[\alpha^{L*},\beta^{L*},\alpha^{R*},\beta^{R*}\right]=x_{t}^{\mathrm{op}}$;31:        Break;32:    **end if**33:**end for** 34:**return** $\left[\alpha^{L*},\beta^{L*},\alpha^{R*},\beta^{R*}\right]$


## 4. Simulations and Experiments

To verify the performance of the proposed method, we simulated the proposed method using accurate data generated based on Python first, and then added different levels of noise to the considered error sources to mimic measurement errors in the real scene and analyze their impacts on the calibration of kappa angle. The practical experiments were also carried out in the actual gaze-tracking system. Since the ground truth of each subject’s kappa angle is unknown, we estimated the 3D gaze of each subject using the calibrated kappa angle and validated the accuracy of kappa-angle calibration through the gaze accuracy.

### 4.1. Computer Simulations

According to the scene of the user sitting in front of the screen and looking at the screen, we generated the required parameters using the Gullstrand–Le Grand eyeball model and the human average [22]. The coordinates of the left corneal centers were randomly generated within a certain range (*x*: from −45 mm to −15 mm, *y*: from −90 mm to −50 mm, *z*: from 350 mm to 650 mm), so the right corneal centers were determined with the constraint that the distance between the 3D corneal centers of the left and right eyes is 60 mm. According to the defined screen, whose corner points were (−200, 262.045317, 250.064495), (200, 262.045317, 250.064495), (200, 69.209034, 20.251162), and (−200, 69.209034, 20.251162), an equal number of on-screen gaze points to the number of corneal centers were randomly generated. Thus, the VA direction was determined using the 3D corneal center and the gaze point. Due to the theoretical range of kappa angle, we set the horizontal component of the kappa angle to 5${}^{{}^{\circ}}$ and the vertical component to 1.5${}^{{}^{\circ}}$. The required OA direction was determined in reverse by using the 3D corneal center, the VA direction of the eyeball, and the kappa angle. The inputs of the model in this paper were 3D corneal centers and OA directions of the left and right eyes, and the output was the calibrated kappa angle. The accuracy and robustness of the model were verified by comparing the predicted and the set kappa angles.

#### 4.1.1. Algorithm Verification

We calibrated the kappa angle by using nine groups of data including 3D corneal centers and OA directions of the left and right eyes. Some constant parameters in our model were set as follows: W=35, K=0.5, and c=1. The iteration range of kappa angles in our simulations was set as follows: $4\leq \alpha^{L}\leq 8$, $-8\leq \alpha^{R}\leq -4$, $0\leq \beta^{L},\beta^{R}\leq 3$. With respect to the iteration termination error of $10^{-9}$, the kappa angles from 10 calibrations are listed in Table 1. “iter_num” represents the number of iterations for which the gaze-constraint error was less than the iteration termination error, and "fun_er" represents its corresponding gaze-constraint error.

**Figure 5:**
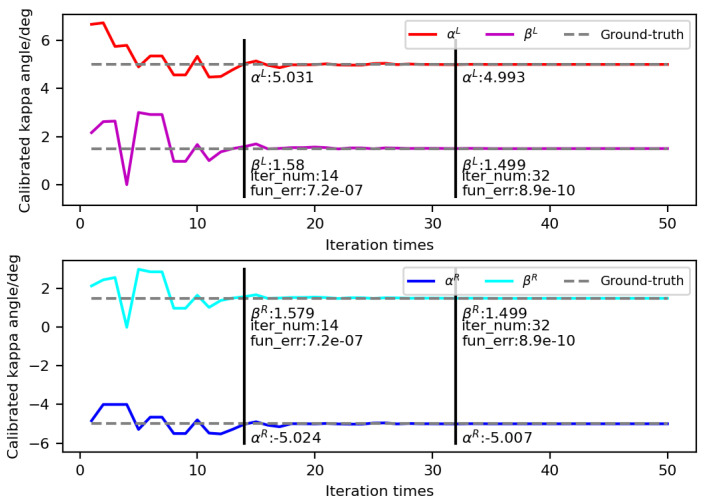
Iteration of kappa-angle calibration in the third round.

Figure 5 shows the iterative process of kappa angles calculated in the third round. The abscissa represents the number of iterations, and the ordinate represents the calibrated kappa angle. As the number of iterations increased, the kappa angles drifted simultaneously toward their true locations. The gaze-constraint error of the 14th iteration was less than $10^{-6}$, and that of the 32th iteration was less than $10^{-9}$. At this time, the values of $\alpha^{L},\beta^{L},\alpha^{R},$ and $\beta^{R}$ were 4.993${}^{{}^{\circ}}$, 1.499${}^{{}^{\circ}}$, −5.007${}^{{}^{\circ}}$, and 1.499${}^{{}^{\circ}}$, respectively, and their errors were less than 0.01${}^{{}^{\circ}}$.

**Figure 6:**
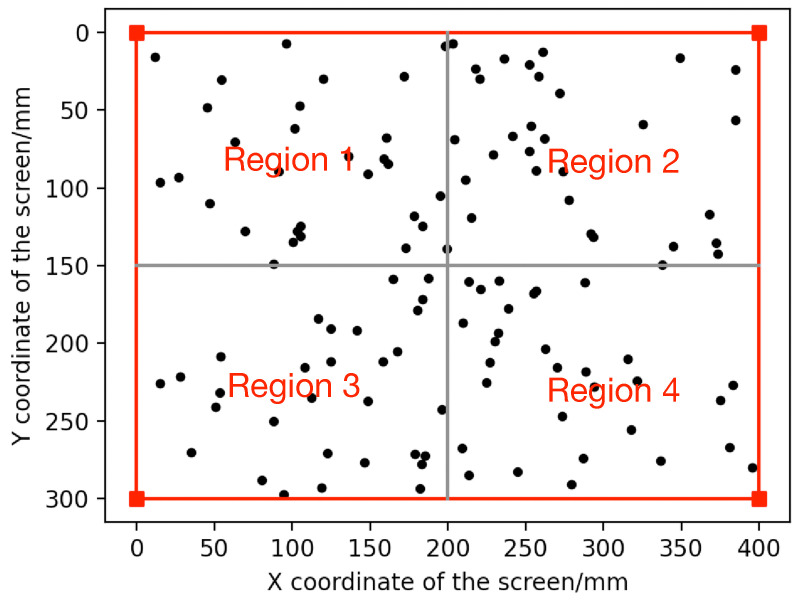
Distribution of gaze points.

(1) The influence of the preset ground truth of the kappa angle: To exclude the influence of the preset ground truth of the kappa angle in the simulation, we analyzed six different sets of ground truth. The horizontal and vertical components of the kappa angle were: [5${}^{{}^{\circ}}$,1.5${}^{{}^{\circ}}$], [4${}^{{}^{\circ}}$,1.5${}^{{}^{\circ}}$], [6${}^{{}^{\circ}}$,1.5${}^{{}^{\circ}}$], [5${}^{{}^{\circ}}$,1${}^{{}^{\circ}}$], [5${}^{{}^{\circ}}$,2${}^{{}^{\circ}}$], and [4${}^{{}^{\circ}}$,0.5${}^{{}^{\circ}}$]. As mentioned in Section 4.1, we first randomly generated nine groups of 3D corneal center coordinates for the left and right eyes within a distance of 350–650 mm, and then randomly generated nine gaze points on the screen. Thus, the VA direction was determined using the 3D corneal center and the gaze point, and the OA direction was calculated in reverse using the different sets of ground truths of the kappa angle. The 3D corneal centers and OA directions were then used for analyzing the influence of the ground truth of the kappa angle. Using the same constant parameters as above, the kappa angle was calibrated. The calibration results using different sets of ground truth are listed in Table 2. It can be seen that no matter what the ground truth of kappa angle is, the error difference of the calibrated kappa angles was not significant, which was less than 0.01${}^{{}^{\circ}}$. Therefore, in the subsequent study, we only analyzed the case where the horizontal and vertical components of the kappa angle were 5${}^{{}^{\circ}}$ and 1.5${}^{{}^{\circ}}$, respectively.

(2) Influence of gaze angle: We divided the screen area into four regions and randomly generated 30 gaze points on each region, as shown in Figure 6. To ensure different gaze angles, we set the same left and right corneal centers corresponding to each gaze point. The 3D corneal centers of the left and right eyes were (−33.62420733, −62.55917489, 508.7146057) and (24.63352113, −76.53688391, 505.449529), respectively. Thereby, the corresponding OA directions of the left and right eyes were generated. Using the data located in the same region, we calibrated the kappa angle, as listed in Table 3. It shows the effectiveness of the proposed method when using different regions of gaze points.

(3) Influence of the amount of data: We also tested the kappa angle calibrated with different amounts of data as input. When 1, 2, 4, 6, 9, 16, 25, 50, and 100 groups of data were taken as the inputs, the respective kappa angles of 10 calibrations were recorded. By comparing them with the ground truth, we calculated the root mean square error (RMSE) of 10 calibrations with different amounts of data, as shown in Table 4. When the data used were less than four groups, the kappa angle could not be exactly iterated due to underdetermined equations. When no less than four groups were used, the kappa angle was accurately calibrated with an error of less than 0.01${}^{{}^{\circ}}$. However, when more than nine groups of data were used, the accuracy improvement was little compared with that of nine groups of data.

In conclusion, the proposed kappa-angle-calibration method can obtain the accurate kappa angle using the accurate corneal centers and OA directions. To obtain the accurate kappa angle quickly, the the amount of data should be considered. When the data reach a certain amount, the accuracy improvement of kappa-angle calibration is little, and the increase in the the amount of data would reduce the operation speed of the algorithm. Moreover, with the increase in the amount of data, the error of the data itself would also be superimposed. Therefore, it is necessary to carry out data filtering according to the actual situation and select the appropriate the amount of data to calibrate the kappa angle.

#### 4.1.2. Error Analysis

Due to system errors and eye-feature-detection errors, gaze-related parameters such as 3D corneal center, 3D pupil center, and iris center often have estimation errors in a real scene. These errors will be transmitted to the OA and VA of the eyeball subsequently. For example, Lu et al. [23] stated that a 0.5 mm error of 3D pupil estimation in *x*- or *y*-axis induced a 3${}^{{}^{\circ}}$ error in gaze angle. Therefore, we analyzed the influences of 3D-corneal-center error and OA error on the kappa-angle calibration, as they are the input of kappa-angle calibration.

Here, in addition to simulating the remote gaze-tracking system described above, we also simulated a head-mounted system for comparison. Four screen corner points were set at (−75, 70, −400), (225, 70, −400), (225, −140, −400), and (−75, −140, −400); and the coordinates of the left corneal centers were generated within certain ranges (*x*: from 5 mm to 10 mm, *y*: from 20 mm to 30 mm, *z*: from 25 mm to 35 mm). Thus, the right corneal centers and OA directions were generated in the same way as the remote system. In combination with the influence of the amount of data, we studied the kappa-angle calibration and the guarantee of a single variable. The same nine groups of data in each system were used for the following error analysis.

**Figure 7:**
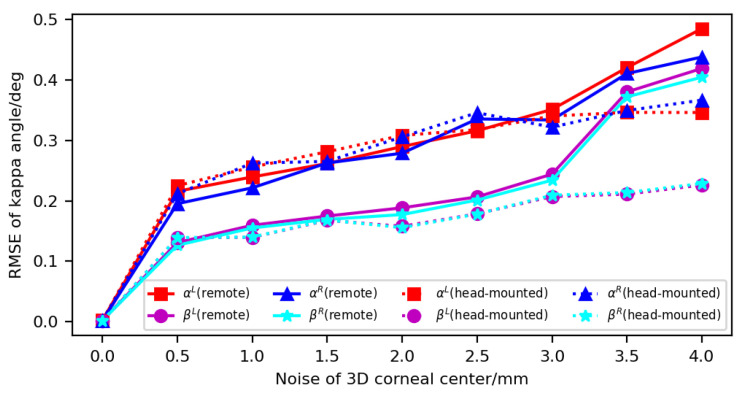
Influence of 3D-corneal-center error on kappa-angle calibration.

**Figure 8:**
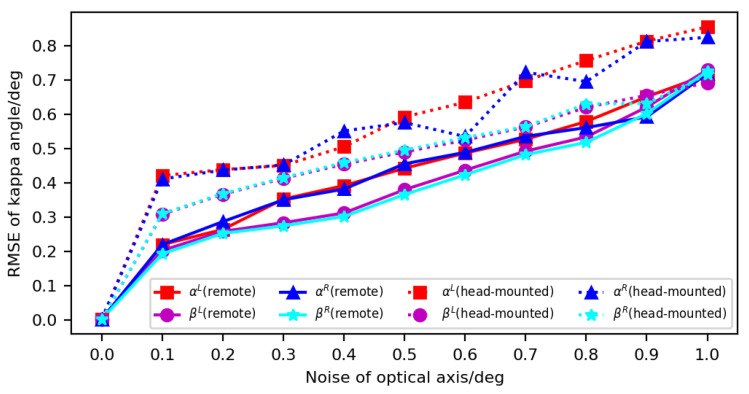
Influence of OA error on kappa-angle calibration.

(1) Analysis of the 3D-corneal-center error: The independent, zero-mean white Gaussian noise was added to the coordinates of the 3D corneal center to simulate its error. When the standard deviation (SD) of the Gaussian noise increased from 0 to 4 mm, the same noise was simultaneously added to the coordinates of the 3D corneal centers of the left and right eyes. For each SD of noise of the 3D corneal center, 100 calibration results of kappa angle were recorded, and the RMSE was calculated. Figure 7 shows the influence of 3D-corneal-center error on kappa-angle calibration. When the SD of the noise changed from 0 to 4 mm, the error of kappa angle increased. For the remote gaze-tracking system, when the SD of the noise was 0.5 mm, the error of the horizontal angle was about 0.2${}^{{}^{\circ}}$, and the error of the vertical angle was about 0.13${}^{{}^{\circ}}$. When the SD of the noise was 4 mm, the errors of the horizontal and vertical angles were larger than 0.4${}^{{}^{\circ}}$. For the head-mounted system, when the SD of the noise was 0.5 mm, the errors were similar to those of the remote system. However, when the SD of the noise was 4mm, the errors were less than those of the remote system. They were about 0.35${}^{{}^{\circ}}$ and 0.23${}^{{}^{\circ}}$ in the horizontal and vertical planes, respectively.

(2) Analysis of OA error: The OA direction in the camera coordinate system has a unique relation to its horizontal and vertical angles in the eyeball coordinate system. To simulate the OA error, we simultaneously added independent, zero-mean white Gaussian noise with a SD of 0–1${}^{{}^{\circ}}$ to the horizontal and vertical angles of the OA directions of the left and right eyes. The SD of the Gaussian noise varied from 0 to 1${}^{{}^{\circ}}$ in steps of 0.1${}^{{}^{\circ}}$, and 100 calibration results of kappa angle under the noise were recorded for each value of SD, and the RMSE was calculated. Figure 8 shows the influence of OA error on kappa-angle calibration. The errors in the head-mounted system were generally larger than those in the remote system. For the remote gaze-tracking system, when the SD of the OA noise was 0.1${}^{{}^{\circ}}$, the errors of the horizontal and vertical angles were about 0.2${}^{{}^{\circ}}$. When the SD of the OA noise was 1${}^{{}^{\circ}}$, the errors of the horizontal and vertical angles reached 0.7${}^{{}^{\circ}}$. For the head-mounted system, when the SD of the OA noise was 0.1${}^{{}^{\circ}}$, the error of the horizontal angle was about 0.41${}^{{}^{\circ}}$, and the error of the vertical angle was about 0.3${}^{{}^{\circ}}$. When the SD of the OA noise was 1${}^{{}^{\circ}}$, the errors of the horizontal and vertical angles were about 0.85${}^{{}^{\circ}}$ and 0.7${}^{{}^{\circ}}$ in the horizontal and vertical directions, respectively.

(3) Discussion: It can be seen from the above analysis that whether in the remote system or the head-mounted system, the OA error was more influential on the kappa-angle calibration than the 3D-corneal-center error. The influence of 3D-corneal-center error on kappa-angle calibration was comparable when used in these two systems. However, when the calibration method was used in the head-mounted system, the influence of OA error was larger than that in the remote system. The fact that both the 3D-corneal-center error and the OA error have a significant impact on the kappa-angle calibration is a universal problem of all kappa-angle-calibration methods [24]. The reasons are as follows: On one hand, as the origin of the eyeball coordinate system, the 3D corneal center is the key to the transformation between the camera coordinate system and the eyeball coordinate system. In addition, the 3D corneal center is the only intersecting point for the OA and the VA, so it plays a vital role in the VA transformation from the OA. On the other hand, the OA direction is the reference direction of VA transformation. If there is an error in the OA direction, the transformed VA direction also has an error. It will appear as a large deviation in the POR on the screen with a large distance between the user and the screen. Therefore, the key to accurately iterating the kappa angle is to ensure the accuracy of the input data.

### 4.2. Practical Experiments

**Figure 9:**
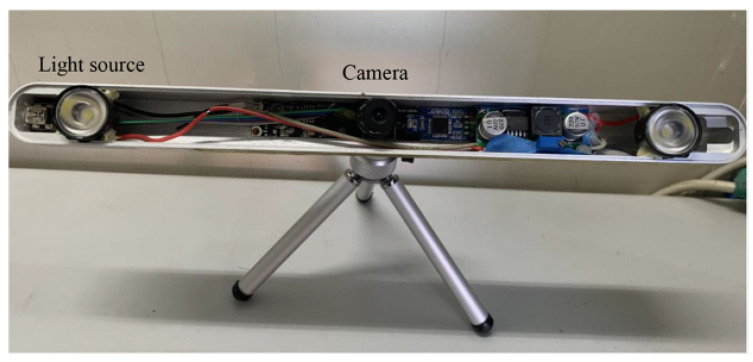
Experimental system.

A single-camera, two-light-source system was used for our practical experiments. A CMOS camera was located in the middle, and two visible light sources were located on both sides of the camera, as shown in Figure 9. The model of the camera was OV7251. Its imaging resolution is 640 × 480, and the pixel size is 2.2 μm. The camera parameters were obtained by the test-range calibration technique, and the positions of light sources were calibrated using the global calibration method [25]. It should be noted that the method proposed in this paper can automatically calibrate the kappa angle without pre-defined calibration points. However, the ground truth of the kappa angle of each subject is unknown. Therefore, the explicit gaze points were used in our experiments, for evaluating the performance of kappa-angle calibration by comparing the POR estimated using the calibrated kappa angle with the explicit gaze point.

The experimental process was as follows: Sixteen test points were preset on the screen, and each subject was asked to sit in front of the screen at a distance between 350 and 600 mm without glasses and look at each test point in turn. During this process, the subject was able to slightly move his head in translation, pitch, and yaw. The facial images were collected by the system’s camera synchronously for subsequent eyeball-feature extraction. The required feature parameters included the iris ellipse parameters and the glints of the left and right eyes. They were extracted by using the eyeball-feature extraction method described in our previous work [26] and then used to estimate the 3D corneal center and the OA direction, where the OA direction was expressed by the iris normal vector.

#### 4.2.1. Performance Analysis

In our experiments, the kappa angles of four subjects were automatically calibrated using the proposed method. The iterative range of horizontal and vertical components of kappa angle was −5${}^{{}^{\circ}}$–5${}^{{}^{\circ}}$. In order to refine the search, the mutation operator *K* was set to 0.01, and the crossover operator *c* was set to 0.1 in consideration of the noise interference. The kappa angle results are listed in Table 5. To verify the accuracy of the kappa angle, the VA direction corresponding to each group of data was calculated using Equation (4). Since our gaze-tracking system has been calibrated [25], the intersection of the VA and the screen was calculated using a point Q on the screen and the screen normal vector $n_{\mathrm{scr}}$—that is, the POR, which is expressed as:

(14) $$S=C+\frac{\left(Q-C\right)•n_{\mathrm{scr}}}{V^{\mathrm{va}}•n_{\mathrm{scr}}}V^{\mathrm{va}}.$$

The average of the PORs of the left and right eyes was taken as the final POR, and the Euclidean distance between the POR and the pre-defined gaze point was regarded as the POR error. The distance from the midpoints of the 3D corneal centers of the left and right eyes to the screen was approximately taken as the distance between the subject and the screen, represented by *D*. Thus, the gaze accuracy expressed by the RMSE is:

(15) $$\theta =\arctan\frac{\sqrt{\frac{1}{N}\sum_{k=1}^{N}{\left(S_{\mathrm{esti},k}-S_{\mathrm{real},k}\right)}^{2}}}{D}.$$

Table 6 lists the gaze accuracy of each subject calculated using the calibrated kappa angle. The proposed method can make the average gaze accuracy reach 1.3${}^{{}^{\circ}}$ in the horizontal direction and 1.34${}^{{}^{\circ}}$ in the vertical direction. Since some researchers approximated the OA as the 3D gaze [27,28], we also calculated the intersection of the OA and the screen (POA), and took the average of the POAs of the left and right eyes as the POR. It can be seen that the gaze accuracy was greatly improved by using the proposed calibration method. Especially when the symbols of the horizontal angles (or vertical angles) of the left and right eyes are the same, the average of the POAs of the left and right eyes had a worse compensation effect on the POR. The POR would bias towards the POA with greater error, resulting in lower accuracy. Therefore, it is necessary to consider the kappa angle to improve the gaze accuracy. The automatic kappa-angle-calibration method proposed in this paper can make the gaze estimation reach the accuracy of less than 2${}^{{}^{\circ}}$, which meets the general application scenario of gaze tracking.

#### 4.2.2. Comparison with Existing Methods

As described in Section 2, there are two main methods for calibrating the kappa angle. One is to calibrate the transformation matrix between the OA and the VA using multiple groups of corresponding OA directions and VA directions obtained from multi-point user calibration, termed the matrix method; the other is to calibrate the horizontal and vertical components of the kappa angle using a single calibration point, termed the angular method. Both methods require explicit calibration points. In this section, we tested these two methods with the same experimental data, and the results are shown in Table 7. For comparison, we also tested the use of constants to represent the kappa angles of the left and right eyes ($\left[\alpha^{L},\beta^{L},\alpha^{R},\beta^{R}\right]=\left[5^{{}^{\circ}},1.5^{{}^{\circ}},-5^{{}^{\circ}},1.5^{{}^{\circ}}\right]$), abbreviated as the constant method.

The gaze accuracy using the matrix method can reach 0.8${}^{{}^{\circ}}$ in the horizontal direction and 1.01${}^{{}^{\circ}}$ in the vertical direction. The matrix method has strong robustness due to the use of multiple known calibration points and has a good transformation effect for the case of large amount of data. However, the explicit calibration process required by this method is relatively complicated, and it is time-consuming for users to look at several on-screen calibration points in turn. The gaze accuracy using the angular method can reach 1.21${}^{{}^{\circ}}$ in the horizontal direction and 1.04${}^{{}^{\circ}}$ in the vertical direction. The angular method uses a single calibration point for the VA transformation, which simplifies the explicit calibration process. However, in order to ensure the gaze accuracy, this method has certain requirements for the selection of calibration points and the robustness of kappa-angle calculation. Although the constant method does not require explicit calibration, it ignores individual differences. In our experiments, the gaze accuracy in both the horizontal and vertical directions was larger than 2${}^{{}^{\circ}}$. The proposed method is essentially to calibrate the kappa angle by obtaining multiple samples through implicit calibration, which has strong robustness. Although the gaze accuracy is not as good as the matrix method and the angular method, it is nevertheless within an acceptable margin of error, as the accuracy of gaze tracking in X- and Y- directions is usually 0.5${}^{{}^{\circ}}$–2${}^{{}^{\circ}}$. It is a development trend of gaze tracking to eliminate explicit user calibration and realize the instant use of gaze-tracking systems, which will promote the wider application of gaze tracking.

## 5. Conclusions

In this paper, an automatic calibration method of kappa angle based on binocular gaze constraint was proposed. The main idea is to use the coplanar constraint of the VAs of the left and right eyes to establish the optimal objective function of the kappa angles of the left and right eyes, and use the differential evolution algorithm to iterate the optimal kappa angle within its theoretical range. Computer simulations verified the feasibility of our method and pointed out that the accuracy of kappa-angle calibration is closely related to the accuracy of 3D-corneal-center estimation and OA reconstruction. The practical experiments showed that the calibrated kappa angle can make the gaze accuracy reach 1.3${}^{{}^{\circ}}$ in the horizontal direction and 1.34${}^{{}^{\circ}}$ in the vertical direction—making our method significantly better than the method without considering the kappa angle. Although the gaze accuracy using the kappa angle auto-calibrated by the proposed method is lower than that obtained by the explicit calibration method, it is still within the acceptable range. Compared with explicit calibration, the automatic calibration method of kappa angle is more in line with the demand for instant use and can provide a better user experience.

It should be noted that the proposed method has the following limitations: (1) We do not consider ethnic differences. The validation experiments were only conducted on a few young Asians. The applicability to other ethnic groups is currently beyond our scope. (2) For special populations such as strabismus or amblyopia, the VAs of the left and right eyes may not be coplanar, which also affects the applicability of the proposed method. (3) The current research status restricts the head rolling of the user. The superposition of head and eyeball movements constitutes the spatial movement of the eyeball. When the head moves in translation, pitch, or yaw, the eyeball parameters will change accordingly, and the head rolling will generate a rotation component of the OA around itself. This rotation component cannot be characterized by analyzing the visual features of the eyeball or calculating the spatial points on the OA, but it will result in changes in the relative position relationship between the OA and the VA, affecting the kappa-angle calibration.

Therefore, expanding upon our research, we are now focusing on the distribution law of kappa angle under natural head movement, especially the influence of head roll on kappa-angle calibration. We attempted to construct head and eyeball postures using some facial features to accurately characterize the OA of the eyeball, thereby ensuring the accuracy of the subsequent kappa-angle calibration, which is also the basis for achieving accurate gaze estimation under natural head movement. In addition, the feasibility analysis of the automatic calibration of the kappa angle considering multiple factors, such as race, gender, age, and health status, and developing it into a universal model, is also our future work.

## Figures and Tables

**Table 1:** Kappa angles obtained from 10 calibrations.

Number of Times	iter_num	$\alpha^{L}{/}^{{}^{\circ}}$	$\beta^{L}{/}^{{}^{\circ}}$	$\alpha^{R}{/}^{{}^{\circ}}$	$\beta^{R}{/}^{{}^{\circ}}$	fun_err
1	33	4.996	1.498	−5.003	1.499	$4.9\times 10^{-10}$
2	30	5.003	1.506	−4.999	1.506	$7.3\times 10^{-10}$
3	32	4.993	1.499	−5.007	1.499	$8.9\times 10^{-10}$
4	33	5.006	1.496	−4.993	1.495	$3.2\times 10^{-10}$
5	40	4.999	1.498	−5.000	1.498	$4.1\times 10^{-10}$
6	35	5.006	1.499	−4.995	1.499	$6.0\times 10^{-10}$
7	35	5.008	1.496	−4.991	1.495	$5.0\times 10^{-10}$
8	35	5.001	1.502	−5.000	1.502	$9.4\times 10^{-10}$
9	39	5.000	1.503	−5.001	1.503	$8.2\times 10^{-10}$
10	27	5.014	1.496	−4.985	1.495	$8.8\times 10^{-10}$

**Table 2:** Kappa angle calibrated from different sets of ground truth.

Ground Truth of Kappa Angle/${}^{{}^{\circ}}$	iter_num	$\alpha^{L}{/}^{{}^{\circ}}$	$\beta^{L}{/}^{{}^{\circ}}$	$\alpha^{R}{/}^{{}^{\circ}}$	$\beta^{R}{/}^{{}^{\circ}}$	fun_err
[5,1.5]	46	5.001	1.499	−4.999	1.499	$1.8\times 10^{-11}$
[4,1.5]	40	4.002	1.501	−3.998	1.501	$8.0\times 10^{-11}$
[6,1.5]	41	6.002	1.501	−5.999	1.501	$4.3\times 10^{-11}$
[5,1]	41	5.000	1.001	−5.001	1.001	$7.7\times 10^{-11}$
[5,2]	45	5.000	2.000	−5.000	2.000	$3.0\times 10^{-11}$
[4,0.5]	41	3.999	0.500	−4.001	0.500	$1.5\times 10^{-11}$

**Table 3:** Kappa-angle calibration results with different gaze angles.

Region	iter_num	$\alpha^{L}{/}^{{}^{\circ}}$	$\beta^{L}{/}^{{}^{\circ}}$	$\alpha^{R}{/}^{{}^{\circ}}$	$\beta^{R}{/}^{{}^{\circ}}$	fun_err
1	38	4.999	1.501	−5.000	1.501	$2.4\times 10^{-11}$
2	34	4.999	1.500	−5.000	1.500	$7.3\times 10^{-12}$
3	40	4.999	1.503	−5.001	1.503	$1.5\times 10^{-11}$
4	39	4.998	1.498	−5.002	1.498	$4.2\times 10^{-11}$

**Table 4:** Influence of amount of data on kappa-angle calibration.

Data Amount	RMSE/${}^{{}^{\circ}}$			
	$\alpha^{L}$	$\beta^{L}$	$\alpha^{R}$	$\beta^{R}$
1	1.186	0.700	1.059	0.619
2	1.014	0.705	0.726	0.722
4	0.004	0.003	0.005	0.003
6	0.003	0.003	0.003	0.003
9	0.002	0.001	0.002	0.002
16	0.002	0.001	0.002	0.001
25	0.001	0.001	0.001	0.001
50	0.001	0.001	0.001	0.001
100	0.001	0.001	0.001	0.001

**Table 5:** Auto-calibrated kappa angles of different subjects.

Subjects	$\alpha^{L}{/}^{{}^{\circ}}$	$\beta^{L}{/}^{{}^{\circ}}$	$\alpha^{R}{/}^{{}^{\circ}}$	$\beta^{R}{/}^{{}^{\circ}}$
1	2.03	2.79	4.41	0.76
2	2.05	0.66	−4.97	0.87
3	0.05	−4.88	−1.22	−2.84
4	0.63	−2.71	−2.91	0.60

**Table 6:** Gaze accuracy of different subjects.

Subjects	Gaze Accuracy/${}^{{}^{\circ}}$	
	Use Auto-Calibrated Kappa Angle	Use the Average of POAs
1	X:0.82, Y:1.29	X:4.27, Y:2.91
2	X:2.05, Y:1.53	X:2.97, Y:2.19
3	X:1.61, Y:1.82	X:2.23, Y:6.86
4	X:0.71, Y:0.71	X:1.26, Y:1.28
Average	**X:1.30, Y:1.34**	X:2.68, Y:3.31

**Table 7:** Comparison of different methods.

Methods	Matrix Method	Angular Method	Constant Method	Proposed Method
Require	Multi-point explicit calibration	Single-point explicit calibration	Without explicit calibration	**Without explicit calibration**
Gaze accuracy/${}^{{}^{\circ}}$	X:0.8, Y:1.01	X:1.21, Y:1.04	X:2.08, Y:2.11	**X:1.30, Y:1.34**

## Data Availability

The data presented in this study are available on request from the corresponding author. The data are not publicly available because they have not been ordered and stored in a clear and manageable form.
